# The needs of family members caring for people living with mental illness: An integrated review

**DOI:** 10.4102/hsag.v30i0.2901

**Published:** 2025-04-16

**Authors:** Keletwaetse Sakwape, Gaotswake P. Kovane, Precious C. Chukwuere, Miriam Moagi, Rorisang Machailo

**Affiliations:** 1 NuMIQ Research Focus Area, Faculty of Health Sciences, North-West University, Mahikeng, South Africa; 2 Department of Nursing, Faculty of Health Sciences, University of Limpopo, Sovenga, South Africa

**Keywords:** caregivers, family, mental illness, needs, support

## Abstract

**Background:**

Family members caring for people living with mental illness (PLWMI) are prone to grapple with caregiver burden. The caregivers often contend with a decline in their mental and physical well-being while executing their role. This exploration aimed to understand the needs of family caregivers of PLWMI.

**Aim:**

An integrative review was conducted to analyse the existing literature on the needs of family caregivers for PLWMI.

**Method:**

The review adhered to the instructions provided by Toronto and Remington, drawing upon the guidance of Whittemore and Knafl. The search used four electronic databases: PubMed, African journals, EBSCOhost and Scopus. After screening 3253 references, 18 studies (10 qualitative, 4 quantitative and 4 mixed methods) were included.

**Results:**

The review identified various needs of PLWMI caregivers, including the need for support (financial, social, peer and psychological), psychoeducation, community acceptance of family caregivers, and comprehensive family caregiver health assessments.

**Conclusion:**

This review recognises the significance of addressing the needs of the caregivers of PLWMI to alleviate the impact of caregiver role strain. Promoting nursing care that recognises the importance of caregiver-oriented practice in healthcare is essential. This can help address the needs of the caregivers, enhance their ability to cope with caregiver role strain and promote a better quality of life.

**Contribution:**

This review may inform policymakers to ensure the need for mental health practice to demonstrate knowledge in caring for caregivers of PLWMI. Furthermore, there is a need to integrate the management of PLWMI with that of the caregivers.

## Introduction

The focus of treating people living with mental illnesses (PLWMI) has shifted from hospitals to their homes over the previous 20 years because of the deinstitutionalisation of mental health services (Addo et al. 2018:2). The deinstitutionalisation of psychiatric patients played a vital role in reducing overcrowding and respecting patients’ autonomy (Silva et al. 2022:102). However, the shift occurred alongside various factors, including the advancement of effective community treatment options like assertive community treatment, the establishment of national programmes that finance housing and community treatment for PLWMI, the impact of the civil rights movements and the significant expenses associated with institutionalising those with mental health conditions (Lamb & Weinberger 2020:175). To this end, most caregiving responsibilities have shifted onto family caregivers rather than mental health professionals.

As a result, families often feel responsible for their loved one’s mental health challenges and feel the need to care for their family members diagnosed with mental illness (Ong, Fernandez & Lim 2021:213). Relational, social and emotional connectedness of family members towards their relatives and a sense of family obligation is the foundation of their nurturing attitude towards caregiving (Sharma, Chakrabarti & Grover 2016:10). Families are driven by a sense of affection, responsibility and commitment to care for their relatives diagnosed with mental illness. However, caring for their loved ones can have many challenges. One of the primary challenges that caregivers face involves the adjustments they must make in their lives to meet the needs of mentally ill family members, particularly those dealing with moderate-to-severe mental illness. The challenges may include halting daily life projects and compromised leisure time (Andrade et al. 2021:2). Another challenge endured by caregivers of people diagnosed with mental illness is courtesy stigma, which literature also refers to as stigma by association (Siddiqui & Khalid 2019:1330). The courtesy stigma of mental illness is the stigma faced by families of PLWMI because of their association with a family member who has a mental illness (Gaolaolwe, Manyedi & Serapelwane 2023:1; Siddiqui & Khalid 2019:1330). The effects of courtesy stigma are reported to be devastating to the families of PLWMI and include feelings of shame, remorse, self-blame, etc. (Azman, Jamir Singh & Sulaiman 2019:462). Therefore, based on empirical evidence, it can be concluded that families of PLWMI experience caregiver burden (Ayalew et al. 2019:1; Hsiao, Lu & Tsai 2020:2746).

In the 1940s, Treudley first used the term ‘caregiver burden’ to describe the negative impact of caring for a chronically sick patient on family dynamics, the caregiver’s health and their quality of life (Chadda 2014:222; The Specialist Forum 2020:12). The causes of caregiver burden include inadequate financial resources, conflicting responsibilities and limited social activities (Liu, Heffernan & Tan 2020:443). Financial and economic constraints are significant factors linked to caregiver burden (Liu et al. 2020:443). Although literature is unequivocal about the causes of caregiver role burden, it remains a dynamic phenomenon, with its intensity influenced by factors such as age, gender, coping strategies, caregiving duration and the cultural values of the caregiver (Chiao, Wu & Hsiao 2015:349). Furthermore, some studies have indicated that social factors such as courtesy stigma can exacerbate caregiver role burden (Kahn et al. 2016:4; Werner et al. 2012:94). While past research has investigated family caregiver burden with their specific needs and coping, the results are frequently inconclusive or contradictory, indicating a lack of a reliable set of practical indicators. This scenario underscores the importance of this integrative literature review (ILR) in providing a more nuanced understanding of the needs of family caregivers caring for PLWMI and uncovering additional influencing factors and trends.

The caregiver burden endured by these families is revealed through the existence of mild emotional and psychosocial difficulties, unfavourable life consequences or major life adjustments that impact the caregivers (The Specialist Forum 2020:12). The World Federation of Mental Health (WFMH) data, as reported by *The Specialist Forum* (2020:12), show that 80% of women who care for PLWMI also have multiple family and work responsibilities. Women in these roles often experience anxiety and depression from caregiving demands. This raises concerns about family caregivers managing stress associated with their responsibilities. Furthermore, research on the needs of these caregivers is limited, which this study aims to address (Ndlovu & Mokwena 2023:2; Stanley, Balakrishnan & Ilangovan 2017:135). Specifically, studies focused on family caregivers of those with mental illness in Botswana are few. The study results could enhance nursing practices by supporting initiatives to meet caregivers’ needs, helping them cope with caregiving pressures. Recognising the specific needs of family caregivers is essential not only for their own well-being but also for the overall effectiveness of mental healthcare. Addressing these needs can foster healthier caregiving settings that enhance the quality of life for caregivers and those they support. Therefore, this study seeks to synthesise the current literature on the needs of family caregivers assisting individuals with mental illness.

## Methods

This ILR adhered to the procedure for conducting an ILR as specified by Toronto and Remington (2020:5), originally adapted from the guidelines by Whittemore and Knafl (2005:548). Whittemore and Knalf (2005:546) define an ILR as a review technique that consolidates existing empirical and theoretical insights to provide a thorough understanding of the subject being investigated. Additionally, the ILR facilitates the incorporation of various methodologies (Toronto & Remington 2020:4). Compared to other review types, such as systematic reviews, the ILR was the most suitable choice for this study. It allowed the researchers to summarise various types of evidence using different methodologies, offering a more comprehensive and inclusive perspective on the needs of family caregivers caring for individuals living with mental illness.

The ILR followed the six-step method outlined by Toronto and Remington (2020), which comprises: (1) formulating the review question, (2) searching and selecting relevant literature, (3) appraising quality, (4) analysing and synthesising data, (5) discussing and concluding and (6) disseminating findings. Conducting the ILR was aimed at synthesising various types of data sources (Papaioannou, Sutton & Booth 2016) about the needs of family members caring for PLWMI. According to Toronto and Remington (2020:5), an ILR synthesises research and extrapolates information from various sources on the subject matter, which facilitates the reviewer’s aptitude to understand a particular phenomenon comprehensively. An ILR best served the purpose of the review because our review necessitated extensive research evidence on the needs of family caregivers of PLWMI. An all-inclusive approach to the literature search was conducted to ascertain a maximum number of eligible primary sources (Whittemore & Knafl 2005:548). To achieve this, the authors started their search on bibliographic databases, then conducted a focused grey literature search and lastly searched reference lists of the primary sources included in the review. The following six steps of the ILR were used in this study as suggested by Toronto and Remington (2020).

**FIGURE 1:**
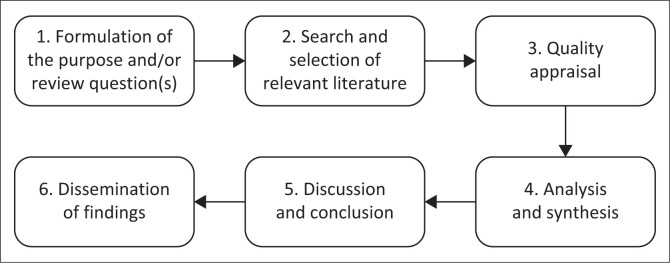
The six steps of the integrative literature review process.

### Formulate review question (problem identification)

The authors started the ILR by identifying a problem, which forms the basis for the ILR (Toronto & Remington 2020:6). The review question for this study was, ‘What is the existing evidence on the needs of family members caring for people living with mental illness?’

### Literature search

Clearly defined literature search approaches are vital as they augment the rigour of any review because biased and incomplete searches led to insufficient databases and a possibility of imprecise findings (Whittemore & Knafl 2005:548). The reviewers systematically approached the literature search. The search took place between September 2023 and December 2023. Moreover, the search was comprehensive, using various electronic databases, ancestry and manual scanning methods through peer-reviewed journals. The bibliographic databases searched are PubMed, African journals, EBSCOhost and Scopus. The search terms were applied across the databases using advanced search options, and MeSH terms were combined using Boolean operators AND and OR. The reviewers derived key search terms from main search terms, which included ‘needs’, OR ‘challenges’, OR ‘experiences’, AND ‘caregivers’, OR ‘family member’, OR ‘families’, AND ‘caring’, AND ‘people’, OR ‘patients’, AND ‘mental illness’. In addition, grey literature was identified using a truncated and structured search strategy from relevant websites of organisations that care for family caregivers such as the Agency for Integrated Care, websites on Google search such as Emerald and databases of peer-reviewed and grey literature (e.g. Google Scholar). Moreover, in this literature search, the reviewers consulted the university librarian concerning the search strategy, the databases and the selection of keywords. The use of a reference librarian benefited the ILR as it augmented the general quality of the search and minimised the risk of bias by enabling a comprehensive and reproducible search procedure (Lawless & Foster 2020:22).

### Inclusion and exclusion criteria

In this ILR, the research articles that were considered for the review included studies published between 2013 and 2023. A decade-long timeframe offers a contemporary perspective on the research landscape within the selected field. Since research develops swiftly, concentrating on this period allows authors to highlight the latest advancements, trends and emerging themes. Previous literature may be obsolete or have been replaced by newer discoveries. The ILR comprised articles from peer-reviewed journals focusing on the needs of family caregivers of PLWMI, published in English. Including literature published in languages other than English can be time consuming and costly because identifying and translating or extracting data from non-English publications can upsurge the time and cost to complete the review (Kugley et al. 2016:32; Rasmussen & Montgomery 2018:2). The study excluded articles and chapters in books that are more than 10 years old and articles from any form of literature review. Incorporating literature review articles can create a dependency on secondary interpretations, potentially diminishing the review’s depth and original analysis. Integrative literature reviews focus on synthesising original research findings instead of analysing prior reviews.

### Data abstraction and synthesis

The PRISMA (Preferred Reporting Items for Systematic Reviews and Meta-Analyses) approach was utilised to review and report article selection (Page et al. 2021:2). The first and last authors independently screened the titles and abstracts of articles against the inclusion criteria. They then advanced eligible publications to the next stage, where abstracts were thoroughly examined. Ultimately, the eligible publications were read in full, and the qualified articles from this set were chosen for the final stage. Any disputes regarding the included articles were resolved through discussion and collegial decision-making before proceeding with the next steps of this integrative review.

### Screening and selection

An in-depth appraisal of the documents was conducted by the authors to critically assess the quality or the validity of the selected articles to determine whether the results are worthy of being included in the study data (Smith et al. 2011:3; Toronto & Remington 2020:7). A critical evaluation of the selected documents was conducted using the Mixed Methods Appraisal Tool (MMAT) (Table 1). The set of questions in the MMAT, covering the qualitative, quantitative and mixed method study designs, makes it suitable for an ILR. The tool is user friendly, thorough, efficient and concise. It features predefined items that facilitate the systematic and transparent examination of key methodological aspects, applying a consistent approach across all included studies (Hong et al. 2018a:464). The first author evaluated all included documents, whereas the second author randomly chose and assessed a selection of them. The articles were assessed according to the MMAT quality criteria, assigning values of yes = 1, no = 0 and cannot tell = 0, while the last value is the aggregate.

**TABLE 1:** Mixed Methods Appraisal Tool version 2018.

Qualitative studies	Quantitative non-randomised studies	Quantitative descriptive studies	Mixed methods studies
Is the qualitative approach appropriate to answer the research questions?	Are the participants representative of the target population?	Is the sampling strategy relevant to address the research question?	Is there an adequate rationale for using a mixed methods design to address the research question?
Are the qualitative data collection methods adequate to address the research question?	Are measurements appropriate regarding both the outcome and intervention (or exposure)?	Is the sample representative of the target population?	Are the different components of the study effectively integrated to answer the research question?
Are the findings adequately derived from the data?	Are there complete outcome data?	Are the measurements appropriate?	Are the outputs of the integration of qualitative and quantitative components adequately interpreted?
Is the interpretation of results sufficiently substantiated by data?	Are the confounders accounted for in the design and analysis?	Is the risk of nonresponse bias low?	Are divergences and inconsistencies between quantitative and qualitative results adequately addressed?
Is there coherence between qualitative data sources, collection, analysis and interpretation?	During the study period, is the intervention administered (or exposure occurred) as intended?	Is the statistical analysis appropriate to answer the research question?	Do the different components of the study adhere to the quality criteria of each tradition of the methods involved?

*Source:* Adopted from Hong, Q.N., Gonzalez-Reyes, A. & Pluye, P., 2018b, ‘Improving the usefulness of a tool for appraising the quality of qualitative, quantitative and mixed methods studies, the Mixed Methods Appraisal Tool (MMAT)’, *Journal of Evaluation in Clinical Practice* 24(3), 459–467. https://doi.org/10.1111/jep.12884

### Data extraction and analysis

The data analysis for this review followed the integrative review framework established by Whittemore and Knafl (2005:548–551). During the critical analysis of the data, the key concepts and viewpoints in the current literature were prudently examined through a critical lens. The first and second authors independently analysed the selected articles using content analysis, whereby words in the text were classified (Grove, Burns & Gray 2021). While coding and integrating data, the researchers identified recurring themes that responded to the review question from both the qualitative and quantitative data. Key findings that correlate to the research aim were highlighted in the article, summarised and listed. The lists of the key findings were grouped according to themes and subthemes. The authors agreed on the themes and subthemes to describe the needs of the family caregivers of PLWMI. The reviewers used insights from a critical and careful analysis of the extant literature to generate new perspectives on the needs and support needed by the family caregivers of PLWMI. Data were extracted into tables and examined for comparisons concerning the review question. Table 2 included authors, year of publication, country, design, population and sampling, purpose and quality appraisal.

**TABLE 2:** Summary of studies included in the review.

Author(s) (year) Country	Study purpose	Methodology (design and methods)	Key findings	MMAT quality criteria
Amini, Jalali and Jalali (2023) Iran	To elaborate on perceived social support by families of patients with chronic mental disorders	Design: Mixed method sequential explanatory. Population: Families of patients with chronic mental disorders Sampling technique: Convenient sampling Sample size: *n* = 346 for quantitative and *n* = 10 for qualitative Data collection: Questionnaires and face-to-face interviews Data analysis: Descriptive and inferential statistics were used in the quantitative phase using SPSS. The qualitative phase was carried out based on the Graneheim and Lundman qualitative content analysis method	Support and acceptance by family, relatives and friends Concerns about support and being understood by society Concerns about social acceptance Need for support (social, financial, emotional, spiritual) Need to be accepted (empathy, attention, free from being judged)	1 1 1 1 1 5
Anokye (2018) Ghana	To explore the needs of family caregivers of people living with mental illness	Design: Exploratory Population: Family caregivers Sampling technique: Purposive Sample size: *n* = 13 Data collection: Semi-structured interviews Data analysis: Miles and Huberman’s framework of thematic content analysis.	Need for physical well-being and support Social need and acceptance Need for social support Need for psychological support	1 1 0 1 1 4
Arkorful et al. (2020) Somalia	To investigate the physio-psychological challenges and needs of family caregivers of people living with mental illness	Design: Qualitative exploratory research Population: Family caregivers of people living with mental illness Sampling technique: Purposive sampling Sample size: *n* = 51 participants Data collection: Semi-structured interviews Data analysis: Miles and Huberman’s framework of thematic content analysis	Need for physiological and physical well-being and support Need for social acceptance and support Need for psychological support	1 1 1 1 1 5
Broady and Stone (2015) New South Wales	To investigate mental health caregivers’ experiences of health and well-being relative to informal caregivers across other contexts To investigate the extent to which mental health caregivers are supported in the vital roles they play for people with mental illness	Design: Quantitative Population: Family mental health caregivers Sampling technique: Not stated Sample size: *n* = 1916 participants Data collection: Online questionnaire Data analysis: Not stated	Mental health caregivers perceived their own health and mental health somewhat more poorly than did other caregivers Need to access mental health services support	0 0 1 1 1 3
De Jesus and Maurice (2020) France	To explore the caregivers’ representations of ‘mental illness’, their challenges and their needs when caring for their ‘mentally ill’ relative To develop an educational programme for empowering the caregivers and supporting positive relationships within the family	Design: qualitative research Population: Caregivers of persons with mental illness Sampling technique: Not stated Sample size: *n* = 15 participants Data collection: In-depth semi-structured interviews Data analysis: Inductive thematic analysis procedure	Social stigma, discrimination and prejudice Emotional distress Social isolation Feelings of love and respite Mental health education and caregiver empowerment Need for social networks Need for peer support (support groups) Need for training Need for conducting home visits by mental health professionals	0 1 1 1 1 4
Iseselo, Kajula and Yahya-Malima (2016) Tanzania	To examine the psychosocial problems and coping strategies of families living with a person with mental illness	Design: Explorative qualitative approach Population: Individual families living with mentally ill persons Sampling technique: Purposive sampling Sample size: *n* = 14 participants Data collection: FGD and in-depth interviews Data analysis: Content thematic analysis	Financial constraints Cost for transport Cost for medication Lack of social support Stigma and discrimination Need for self-help groups Need for psychoeducation	1 1 1 1 1 5
Kumar and Das (2017) India	To identify the perceived rehabilitation needs of caregivers of PWLMI	Design: Descriptive cross-sectional study Population: Caregivers of persons with mental illness Sampling technique: Purposive sampling Sample size: *n* = 100 caregivers Data collection: Socio-demographic information sheet, and self-structured perceived rehabilitation needs questionnaire Data analysis: Descriptive statistics	Needs related to medical facilities, entertainment and leisure time activities, family life and self-care were more emphasised by the caregivers as compared to other rehabilitation needs. Needs related to the development of the right attitude in the care of the PLWMI and obtain peer or community support in the care of the PLWMI were reported equally important by the caregivers Need for psychoeducation	1 0 0 1 1 3
Marimbe et al. (2016) Zimbabwe	To explore the impact of caring for a family member with a mental disorder, as well as their coping strategies and needs	Design: Mixed methods Population: Adult family members who were the primary caregivers of persons diagnosed with mental disorders Sampling technique: Purposive sampling Sample size: *n* = 31 family members (9 in the in-depth interviews and 22 in the FGDs) Data collection: Structured individual in-depth interviews, focus group discussions, SSQ 14 Data analysis: Thematic approach with NVivo 8 for qualitative data. Quantitative data obtained from the SSQ were captured using Epi Info 7 and analysed using SPSS, version 16.	Physical harm or illness Psychological or emotional impact Financial or material impact Need for financial assistance Need for support groups Need for training to deal with psychological problems Need for emotional support	1 1 1 0 1 4
Mark (2013) Rhode Island	To explore the care giving experience of people who serve as caregivers for mentally ill family members	Design: Qualitative, exploratory Population: Caregivers for a relative suffering from a chronic and severe mental illness Sampling technique: Not stated Sample size: *n* = 14 Data collection: Semi-structured interviews Data analysis: Not stated	Caregiving responsibilities Caregiving and its impact Biggest change in caregiver’s life Need for support (psychological, financial and social)	1 1 1 0 0 3
Molepo and Mfidi (2020) South Africa	To gain insight into the lived experiences of young people living with MHCUs	Design: Qualitative descriptive phenomenological research Population: Young people who live with MHCUs Sampling technique: Purposive sampling Sample size: *n* = 10 participants Data collection: Unstructured interviews Data analysis: Content analysis	Psychological effects ◦ Ashamed and embarrassed ◦ Feeling of despair ◦ Fear and guilt Added responsibility ◦ Burden of care ◦ Non-adherence to treatment Effects on school performance ◦ Poor academic performance and progress ◦ Resulted dropouts Coping strategies and support ◦ Individual coping mechanisms ◦ Being accommodating ◦ Avoid and ignore ◦ Smoking and alcohol abuse ◦ Support systems (family, community) ◦ Economic factors ◦ Need for psychoeducational programmes	1 1 1 1 1 5
Moudatsou et al. (2021) Greece	To explore the views of mental health professionals regarding the needs of the informal caregivers of patients with chronic psychotic syndrome	Design: Qualitative case study Population: Mental health professionals who work in public healthcare services Sampling technique: Purposive sampling Sample size: *n* = 12 health professionals Data collection: Semi-structured, face-to-face interviews Data analysis: Framework analysis	Impact of caring on caregivers’ lives ◦ Financial and professional burdens ◦ Social constraints ◦ Physical and mental deterioration Caregivers’ needs ◦ Economic support ◦ Psychosocial support ◦ Information needs Recommendations for better care ◦ Meeting the caregivers’ needs ◦ Improvement of services	1 1 1 1 1 5
Nair et al. (2018) India	To assess the need for and feasibility of initiating microfinance groups for the caregivers of persons with mental disability	Design: Mixed methodology design with cross-sectional survey. Population: Families and women caregivers in each household for a person with mental disability Sampling technique: Random sampling and semi-structured interviews Sample size: *n* = 10 households/women caregivers Data collection: GHQ-5 and Symptoms and Others checklist and the women caregiver was interviewed using qualitative needs assessment schedule and Perceived Social Support Scale. Data analysis: The quantitative socio-demographic data were entered in SPSS 21 (IBM, version) and were analysed using descriptive statistics, while thematic analysis was used to analyse the quantitative data.	Financial burden Capacity of the caregiver Community resources Unavailability of social support Financial needs	1 1 1 0 1 4
O’Neill et al. (2022) Northern Ireland	To identify how services could support caregivers’ well-being and the recovery of those who benefit from their care	Design: Not specified Population: Caregivers of a family member with a significant mental health problem Sampling technique: Purposive sampling Sample size: *n* = 11 Data collection: Face-to-face, semi-structured interviews Data analysis: Thematic analysis	Sources of stress ◦ Becoming a caregiver ◦ The challenge of family obligations and multiple roles ◦ Navigating difficult relationships and responsibilities ◦ Engaging with services The impact on health and well-being ◦ Impact on mental well-being and mental health of the caregiver ◦ Need for support and respite but no-one else can help ◦ Coping with stress	0 1 1 1 1 4
Phoeun et al. (2023) Cambodia	To explore the psychosocial stressors and concerns of relative caregivers of people with severe mental illness	Design: Convergent mixed methods Population: Relative caregivers of patients with mental illness Sampling technique: Convenience sampling Sample size: 37 participants for the FGD (6–9 per group), and 115 participants provided quantitative survey data Data collection: Semi-structured focus group discussions and quantitative interview battery Data analysis: Qualitative data were analysed using iterative process to produce themes. Quantitative data were analysed statistically	Financial burden Erratic behaviour of patients Social alienation Somatic and emotional symptoms Need for peer support groups Need for mental health assessment Need for psycho-therapeutic supports Need for provision of in-home consultation to improve safety	1 1 1 1 1 5
Shamsaei, Cheraghi and Esmaeilli (2015) Iran	To explore the challenges with which the family caregivers of individuals with chronic mental illness have to contend	Design: Phenomenology Population: Family caregivers of individuals with chronic mental illness Sampling technique: Purposive sampling Sample size: 16 participants Data collection: Qualitative interviews Data analysis: Colaizzi’s phenomenological method	Stress and emotional distress Need for education and information Socioeconomic effects and support Need for social support Physical strain	1 1 0 1 1 4
Silaule, Gloria and Adams (2023) South Africa	To establish the extent of subjective and objective burdens among informal caregivers of people with severe mental disorders	Design: Descriptive quantitative cross-sectional design Population: Informal caregivers of people with severe mental disorders Sampling technique: Non-probability convenience sampling Sample size: 170 Data collection: Structured interviews Data analysis: Descriptive analysis using Stata v15	The informal caregivers who resided with care recipients reported significantly higher objective burden. The global burden scores revealed considerable burden among the informal caregivers. Need to integrate the assessment of burdens among informal caregivers of people with SMDs in routine clinical practice	1 0 0 1 1 3
Vukeya, Temane and Poggenpoel (2022) South Africa	To describe family members’ experiences caring for a sibling with mental illness	Design: Qualitative, exploratory, descriptive and contextual research design Population: Family members caring for a sibling with mental illness Sampling technique: Purposive sampling Sample size: Eight Data collection: In-depth phenomenological interviews Data analysis: Thematic coding	Family members find caring for a sibling with mental illness to be overwhelming. ◦ Unpredictability of the mental illness ◦ Relapse because of noncompliance ◦ Siblings displayed aggressive outbursts Family members experience emotional instability as a result of caring for their sibling with mental illness. ◦ Fear related to the sibling’s behaviour, hopelessness, helplessness and concern over the sibling’s safety ◦ Family disequilibrium extending to community members ◦ Financial instability related to the cost of caring for a sibling with mental illness Family members need support in caring for their sibling with mental illness. ◦ Need for home visits ◦ Need for more information about mental illness ◦ Need for a place of safety for the sibling with mental illness	1 1 1 1 1 5
Zacharis, Lyrakos and Zisi (2020) Greece	To explore the connection between physical activity and mental health status in family caregivers of patients with mental illness	Design: Cross-sectional design Population: Family members of patients with mental illness Sampling technique: Not defined Sample size: 300 Data collection: Structured questions Data analysis: SPSS 24	Increased physical activity and exercise are important for caregivers’ mental health and caring role.	0 1 1 1 1 4

*Source:* Adapted from Hwang, J.J., Donnelly, T.T., Raffin Bouchal, S. & Davidson, S., 2023, ‘Factors influencing access to nonpharmacological interventions for community-dwelling seniors with mild-to-moderate dementia: An integrative review’, *Journal of Psychiatric and Mental Health Nursing* 30(6), 1054–1081.

Note: yes = 1, no = 0, cannot tell = 0) 1 2 3 4 5 Σ; Please see full reference list of this article: https://doi.org/10.4102/hsag.v30i0.2901.

MMAT, Mixed Methods Appraisal Tool; SPSS, Statistical Package for Social Sciences; FGD, focus group discussions; PLWMI, people living with mental illness; SSQ, Shona Symptom Questionnaire; GHQ, General Health Questionnaire.

### Ethical considerations

Ethical clearance to conduct this study was obtained from the North-West University Health Research Ethics Committee (NWU-HREC) on 04 May 2024 (ethic no.: NWU 0017823A1).

## Review findings

### Study characteristics

**FIGURE 2:**
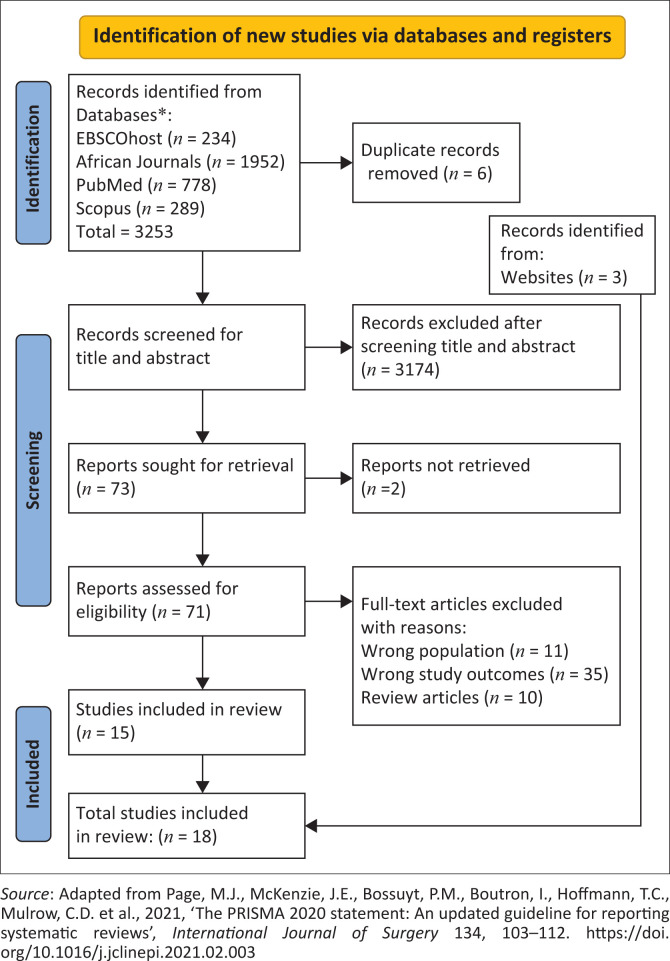
Flow diagram showing the inclusion and exclusion process of the integrative literature review.

The 18 studies included in the ILR covered 2013–2023. The screening process of the ILR is summarised in Figure 2. The focus of the articles is on the caregivers of PLWMI. Of the 18 studies, 55.6% (*n* = 10) used a qualitative design, 22.2% (*n* = 4) used a quantitative research design and 22.2% (*n* = 4) used a mixed methods design. Moreover, the studies included in this review were conducted in lower-income countries (11.1%; *n* = 2), lower-middle-income countries (27.8%; *n* = 5), upper-middle-income countries (27.8%; *n* = 5) and high-income countries (33.3%; *n* = 6). An integrated summary of the characteristics of the studies included in the ILR is presented in Table 2.

## Results and synthesis of the findings

The research studies used various assessment approaches to validate the collected data. The studies depended on face-to-face interviews, structured questionnaires and interviews, semi-structured interviews, in-depth interviews, focus group discussions and unstructured interviews. The aforementioned approaches were used to collect data on the caregivers of PLWMI’s experiences and needs in caring for their loved ones.

Appraisal of the studies included in this ILR yielded four themes and subthemes related to the needs of family caregivers of PLWMI, which were discussed below: (1) need for support (subthemes – social needs and support, psychological needs and support, physical needs and support, financial needs and support and need for peer support); (2) need to be accepted; (3) need for psychoeducation and (4) need for assessment (see Table 3).

**TABLE 3:** Summary of themes and subthemes relating to the needs of family caregivers.

Themes	Sub-themes
Need for support	1.1. Social needs and support 1.2. Psychological needs and support 1.3. Physical needs and support 1.4. Financial needs and support 1.5. Need for peer support
Need to be accepted or not to be judged	-
Need for psychoeducation	-
Need for assessment	-

### Theme 1: Need for support

It is impossible to overstate the importance of support networks in preventing the negative impacts such as economic difficulties, physical and emotional strain on PLWMI family care (Addo et al. 2018:2; Molepo & Mfidi 2020:9). Support is essential for helping caregivers of PLWMI manage the stress of their care responsibilities, as indicated by four studies (Amini, Jalali & Jalali 2023:7; Broady & Stone 2015:328; O’Neill et al. 2022:2037). Amini et al. (2023:5) state that a critical component of the sustainability of PLWMI caregivers is the availability or lack of assistance. In a study by Broady and Stone (2015:328), family caregivers for mental health issues reported that they needed help. Considering the relatively low ratings of their physical and mental well-being, the caregivers in the previously stated survey emphasised how urgently they needed access to support services. In a different study by O’Neill et al. (2022:2037), caregivers emphasised the need for assistance and respite to help them deal with the stress of caring. This can be demonstrated by asking for help and taking time off from caring responsibilities.

#### Subtheme 1.1: Social needs and support

In a study by Amini et al. (2023:1-8), the concept of support and society comprehension was demonstrated using interview analyses. The caregivers of PLWMI frequently identified the main challenges in the caregiving journey as issues related to social support, including welfare, health programmes and social contacts. The results of a study by Anokye (2018:335) showed that the social requirements of family caregivers and their relatives diagnosed with mental illness are not substantially different because of the cultural stigma associated with mental illness and the social obstacles that emerge from it. In the previously mentioned study, caregivers identified social demands, including the need for social affection. This encompasses the importance of promoting and supporting interactions within their broader social network, allowing society to embrace and express love towards them and facilitating easier participation in social activities. Arkorful et al. (2020:14) found that participants desire social connectivity. This refers to caregivers’ perceptions of their relationships with others, both within and outside the caregiving setting. It encompasses the quantity and quality of their social interactions, their sense of belonging and the support they obtain from their social networks. According to the caregivers, the stress of caregiving tasks interfered with their social lives and commitments, making it difficult for them to fulfil their social duties (Anokye 2018:335; Arkorful et al. 2020:14; Mark 2013:41). Caregivers report that having social support is essential when caring for a relative who is dealing with a persistent mental illness (Shamsaei et al. 2015:5). They claim that because other family members are unaware of the stress they are under while taking care of their loved ones, they receive less help.

#### Subtheme 1.2: Psychological needs and support

Five studies reported the family caregivers’ psychological needs and the support they need to cope with the strain of caregiving (Anokye 2018:336; Arkorful et al. 2020:13; Marimbe et al. 2016:5; Moudatsou et al. 2021:10; Phoeun et al. 2023: 444).

Results from research involving family caregivers of PLWMI show that the participants’ main urgent psychological need was a good attitude towards their relatives diagnosed with mental illness, leading to a better state of mind (Anokye 2018:336; Arkorful et al. 2020:13; Marimbe et al. 2016:5). The participants also stated that they needed love and affection as well as counselling to help them achieve a stable mental state so they could fulfil their role as caregivers. According to the aforementioned studies, family caregivers report experiencing psychological difficulties such as stress, worry and depression because of the strain and complications involved in providing care for their relatives diagnosed with mental illness. It can thus be concluded that unmet psychological needs amplify caregiver burden. Inadequate means to express feelings can overwhelm caregivers because of stress and other psychological demands.

According to Moudatsou et al. (2021:10), participants reported the need for psychological support as a priority for the family caregivers of PLWMI because of the difficulties in coping and adapting because of issues relating to mental illness, social stigma and patient behaviour. The above findings are supported by a study conducted by Phoeun et al. (2023:444), which highlights psycho-therapeutic support as one of the priority interventions needed by family members caring for PLWMI.

#### Subtheme 1.3: Physical needs and support

According to the body of research, family caregivers of PLWMI have reported dealing with a variety of physically excruciating difficulties while providing care, placing them in a position of equal vulnerability with their patients (Anokye 2018:335; Arkorful et al. 2020:9; Shamsaei et al. 2015:5). The caregivers encounter numerous physical challenges, such as trouble sleeping, severe headaches, fatigue and chest pains. Research investigating caregivers’ support and assistance requirements revealed various physical health challenges. They expressed the need for additional caregiving support to help them plan for adequate sleep and access medical care (Anokye 2018:335; Arkorful et al. 2020:9). Some participants also emphasised the importance of providing accommodations for their patients after they are discharged from the hospital. Adding exercise programmes to support family caregivers, as suggested by Zacharis et al. (2020:853), is crucial. Studies demonstrate the significance of physical activity and exercise for family caregivers’ mental and physical well-being.

#### Subtheme 1.4: Financial needs and support

According to published research, one of the main obstacles facing the majority of family members who look after their PLWMI is financial constraints (Shamsaei et al. 2015:5). According to the author, several caregivers relied on charity and loans from friends and family to get by. This was corroborated by a study by Nair et al. (2018:40) in which the family caregivers stated that they required financial support. To assist their families and achieve financial independence, they wanted to participate in any self-help groups focused on microfinance. In other research, family caregivers asked for financial support from the government or well-wishers, indicating that having or not having financial support was essential to the family caregivers’ ability to continue their work (Amini et al. 2023:8; Marimbe et al. 2016:5).

#### Subtheme 1.5: Need for peer support

Three studies described the need for peer support to deal with the strain of caregiving as one of the key necessities for the family caregivers of PLWMI (De Jesus & Maurice 2020:14; Iseselo et al. 2016:9; Marimbe et al. 2016:5). In a study conducted by De Jesus and Maurice (2020:14), most family caregivers conveyed the significance of networks to enable them to share about their burden in caregiving and learn about mental health issues. Social support groups can take various forms and aim to foster social interaction for caregivers and their loved ones facing mental illness, ultimately enhancing their quality of life (Iseselo et al. 2016:9).

### Theme 2: Need to be accepted or not to be judged

Concern about being accepted is one of the central issues for families caring for their loved ones with chronic mental illnesses (Amini et al. 2023:5). The participants in the aforementioned study indicated the need for acceptance, empathy and attention, which can enhance their endurance in their caregiving role. Other studies revealed the need for social affection by the family, which they described as the need to be shown love and acceptance by society (Anokye 2018:332; Arkorful et al. 2020:16). The participants expressed that their predicament, which emanates from social stigma associated with mental illness, puts them in dire need for more social attention.

### Theme 3: Need for psychoeducation

Eight studies reported the need for education to family caregivers of PLWMI. The family caregivers expressed their need for education and awareness about mental illness (De Jesus & Maurice 2020:12; Kumar & Das 2017:6; Vukeya et al. 2022:7). The caregivers expressed the need for information on the diagnosis, management and prognosis of the patient’s mental condition (Shamsaei et al. 2015:5). They reported the need for education on practical aspects of care and guidance on how to relief the strain of caregiving, which can benefit both the caregiver and their relative diagnosed with mental illness (Molepo & Mfidi 2020:9; Moudatsou et al. 2021:10). According to Iseselo et al. (2016:8), caregivers indicated their interest in acquiring more insight into the genesis of mental illness, which can avert stigma and discrimination and its associated stress. In a study conducted by Marimbe et al. (2016:5), the caregivers reported deficient knowledge about their relatives’ mental conditions and requested to be trained to deal with caregiving psychological effects.

### Theme 4: Need for assessment

Literature indicates that health assessment of the family care providers should be of primary concern to mental health professionals (Phoeun et al. 2023:443). Studies recommend that family care providers’ needs assessment should be conducted as they accompany their relatives for treatment at health facilities (Phoeun et al. 2023:443; Silaule et al. 2023:9). The caregivers of PLWMI should be conducted to evaluate various aspects of the caregiver’s life, including their subjective burdens and coping needs (De Jesus & Maurice 2020:20; Vukeya et al. 2022:7). According to De Jesus and Maurice (2020:20), findings from the caregivers’ assessments would further assist the health professionals in identifying the strategies and interventions that can be employed to assist the caregivers meet their needs. Assessment of caregivers can aid in identifying caregiver-specific needs, thus enabling targeted interventions to alleviate caregiver burden.

## Discussion

While the family unit continues to be the foundation and strength of PLWMI, family caregivers encounter several challenges as their care transition. To effectively prevent the detrimental impacts on family members who are caring for PLWMI, support networks and services are required (Amini et al. 2023:8; O’Neill et al. 2022:2033). Supporting PLWMI is critical to avoiding caregiver burnout and enhancing their quality of life and overall well-being (Von Kardorff et al. 2015:6).

Availability of support or lack thereof is a critical factor in determining the sustainability of family caregivers, as support enhances the caregivers’ quality of life (Amini et al. 2023:8; Vukeya et al. 2022:7). Access to extra help and support groups is frequently linked to the capacity to manage the stress of providing care (Sharif et al. 2020:9). According to Ntsayagae, Myburgh and Poggenpoel (2019:4), social support is thought to be protective against the psychological challenges faced by family caregivers of PLWMI. Therefore, it is essential to develop support programmes and policies that address the well-being of family members caring for PLWMI.

The findings of our research reveal a significant lack of adequate support for families of PLWMI in Africa. This shortfall stems from many African countries’ economic challenges, raising considerable concern for the PLWMI caregivers’ well-being (Opondo et al. 2020:2; Verity et al. 2021:7). Thus, as postulated by Hsiao et al. (2020:2746), these families bear the burden of caregiving with inadequate support. Contrary to the foregoing, developed nations such as Western countries have made significant strides in supporting PLWMI. In these countries, the much-needed support for caregivers of PLWMI is provided through respite care, halfway homes and community-based centres, which are used to take care of PLWMI and relieve their families from the caregiving burden (Bïlïr 2018:565). Moreover, families of PLWMI require financial, social, psychological and spiritual support that would provide them with enough resources to care for their loved ones (O’Neill et al. 2022:2033). Health workers should promote support for the families of PLWMI. This may include inter-alia, advocating for the establishment of respite care centres and provision of both financial and psychosocial support to ease the caregiving burden. Family caregivers frequently experience overwhelming challenges because of their patients’ diagnoses. Collaborating with mental health professionals, support groups, psychotherapy and group therapy for caregivers facing similar situations can help address this issue. Financial aid is crucial in alleviating the economic pressures related to treatments, including regular check-ups and other therapeutic activities that place substantial financial strain on caregivers. Consequently, providing external financial resources is vital to ease their burdens.

One of the primary concerns in family care of PLWMI is the fear of not being accepted. Family caregivers of PLWMI feel neglected and ostracised (Gaolaolwe et al. 2023:5). Families frequently endure unwarranted stigmatisation from society, often being categorised as relatives of individuals with mental illness simply for providing care to these patients. This detrimental labelling, along with stereotypes and discrimination, not only damages their reputation but also exacerbates the already substantial burdens faced by caregivers. It is imperative that society actively challenges these biased perceptions and offers support to these families, thereby recognising their essential role in the welfare of those they care for. Thus, mental health professionals, like social workers, psychiatric nurses, etc., should promote acceptance for families of individuals with mental illness. Freeing caregivers from courtesy stigma is vital for coping with the challenges of care, achievable through public health education and awareness campaigns (Gaolaolwe et al. 2023:5). The community’s acceptance of caregivers can enable them to form stronger social networks where they can exchange information and support related to mental health (De Jesus & Maurice 2020:11).

In addition, guidelines and policies on caregiving should emphasise the importance of providing care information to families of PLWMI (Moudatsou et al. 2021:10). Caregivers must learn about their relative’s mental health diagnosis and its management for improved care (Chadda 2014:225; Shamsaei et al. 2015:5). Research underscores the importance of health professionals dedicating time to caregivers to comprehend their struggles and create educational approaches addressing the psychological challenges they encounter during their caregiving experience (De Jesus & Maurice 2020:12; Gaolaolwe et al. 2023:5; Marimbe et al. 2016:5; Von Kardorff et al. 2015:6; Vukeya et al. 2022:7).

Mental health professionals must conduct timely assessments for families of PLWMI to promptly identify those facing caregiver burden. The results of these assessments can help health workers create suitable interventions and support tailored to these families, enabling them to manage the challenges of caregiving (Chadda 2014:225; Phoeun et al. 2023:443). Assessing family caregivers helps identify their strengths and weaknesses, guiding effective interventions and support (Foster et al. 2016:147). Therefore, it is essential that caregivers accompanying their relatives diagnosed with mental illness for consultations also have their well-being assessed. Moreover, caregivers’ assessments can be carried out through home visits by mental health professionals (Vukeya et al. 2022:7).

### Implications and recommendations

This ILR has implications for mental healthcare providers. The review sheds light on the needs of family caregivers of PLWMI. It highlights the significance of support – financial, social, physical and psychological – as well as psychoeducation and assessments that mental health and welfare personnel should recognise. The review also emphasises the importance of understanding caregivers’ perspectives and experiences, calling for more in-depth exploration using comprehensive study designs. Healthcare providers need to take note of the findings and intensify public education to raise awareness of mental health issues for the caregivers of PLWMI to feel free to socialise without fear of being discriminated against. Furthermore, it is essential to integrate the management of family caregivers with that of their loved ones. Mental health practice should demonstrate knowledge in caring for caregivers of PLWMI to help them cope with the burden of caregiving, which can be achieved through addressing their needs. Additionally, there should be continuous education and training for family caregivers to enhance continuity of care for PLWMI at home across their lifespan. This necessitates policymakers to create legislation mandating the support of caregivers of PLWMI, including the development of guidelines to assist family caregivers.

### Limitations

This integrative review used a structured outline to effectively summarise research results from various study designs, providing valuable insights into the needs of PLWMI caregivers. The review authors acknowledge limitations, such as potential language bias in excluding non-English studies and possibly selecting biased studies. Additionally, the review excluded grey literature, articles older than 10 years, books and chapters in books, which might lead to authors missing valuable rich data that can enrich the ILR. The review also identifies limitations such as a lack of literature on the needs of family caregivers of PLWMI. Most studies included in this review focused on the challenges and experiences of family caregivers, with only two studies specifically exploring their needs. The review also acknowledges a deficiency in data saturation, with most findings being based on just a few studies. It highlights the need for further investigation into areas such as the impact of cultural beliefs on caregivers’ needs. Therefore, further studies are needed to explore the needs of families of PLWMI in their specific contexts.

## Conclusion

This review explored the needs of family caregivers of PLWMI. The review sums up a comprehensive indication of the needs of caregivers of PLWMI emanating from caregiver role strain and how addressing the needs can assist them cope with the strain of caring. The review outlines the thematic findings on the needs of family caregivers of PLWMI, which includes the need for support (financial, social, physical peer and psychological support), the need to be accepted, the need for psychoeducation and the need for assessment. The review discovered that caregiver burden is a significant issue in the caregivers of PLWMI and needs to be managed through addressing the family caregivers’ needs. It is imperative that mental health nurses identify the needs of the family caregivers in caring for PLWMI and address them to help them cope and improve their quality of life. Alleviating caregiver role strain should be the central focus. A caregiver-oriented care practice could be employed to target caregiver role strain and address the caregivers’ needs. In light of this study’s findings, the researchers recommend developing caregiver support policies and guidelines to guide policymakers on the support they should provide to address the needs of families of PLWMI.

## References

[CIT0001] Addo, R., Agyemang, S.A., Tozan, Y. & Nonvignon, J., 2018, ‘Economic burden of caregiving for persons with severe mental illness in sub-Saharan Africa: A systematic review’, *PLoS One* 13(8), 1–12. 10.1371/journal.pone.0199830 PMC6084810 30092073

[CIT0002] Amini, S., Jalali, A. & Jalali, R., 2023, ‘Perceived social support and family members of patients with mental disorders: A mixed method study’, *Frontiers in Public Health* 11(1), 1–10. 10.3389/fpubh.2023.1093282 PMC9939439 36815153

[CIT0003] Andrade, J.J.D.C., Silva, A.C.O., Frazão, I.D.S., Perrelli, J.G.A., Silva, T.T.D.M. & Cavalcanti, A.M.T.S., 2021, ‘Family functionality and burden of family caregivers of users with mental disorders’, *Revista Brasileira de Enfermagem* 74(5), 1–9. 10.1590/0034-7167-2020-1061 34468548

[CIT0004] Anokye, R., 2018, ‘The needs of family caregivers of people living with mental illness: A social workers’ assessment’, *Practice* 30(5), 323–339. 10.1080/09503153.2018.1436702

[CIT0005] Arkorful, V.E., Mohamed, A.A., Jama, O., Pokuaah, S., Basiru, I., Hammond, A. et al., 2020, ‘Caregivers of mentally ill patients: A cross-sectional need-based assessment of social workers in post-conflict Somalia’, *Practice* 32(1), 21–41. 10.1080/09503153.2019.1620200

[CIT0006] Ayalew, M., Workicho, A., Tesfaye, E., Hailesilasie, H. & Abera, M., 2019, ‘Burden among caregivers of people with mental illness at Jimma University Medical Center, Southwest Ethiopia: A cross-sectional study’, *Annals of General Psychiatry* 18, 1–11. 10.1186/s12991-019-0233-7 31285750 PMC6591984

[CIT0007] Azman, A., Jamir Singh, P.S. & Sulaiman, J., 2019, ‘The mentally ill and their impact on family caregivers: A qualitative case study’, *International Social Work* 62(1), 461–471. 10.3109/09638237.2015.1124395

[CIT0008] Bïlïr, M.K., 2018, ‘Deinstitutionalisation in mental health policy: From institutional-based to community-based mental healthcare services’, *Hacettepe Sağlık İdaresi Dergisi* 21(3), 563–576.

[CIT0009] Broady, T.R. & Stone, K., 2015, ‘“How can I take a break?” Coping strategies and support needs of mental health caregivers’, *Social Work in Mental Health* 13(4), 318–335. 10.1080/15332985.2014.955941

[CIT0010] Chadda, R.K., 2014, ‘Caring for the family caregivers of persons with mental illness’, *Indian Journal of Psychiatry* 2014(56), 221–227. 10.4103/0019-5545.140616 PMC4181176 25316932

[CIT0011] Chiao, C.Y., Wu, H.S. & Hsiao, C.Y., 2015, ‘Caregiver burden for informal caregivers of patients with dementia: A systematic review’, *International Nursing Review* 62(3), 340–350. 10.1111/inr.12194 26058542

[CIT0012] De Jesus, N.S. & Maurice, A., 2020, ‘Exploring mental health caregivers’ caregiving experiences in France’, *The Journal of Mental Health Training, Education and Practice* 15(4), 207–221. 10.1108/JMHTEP-06-2019-0031

[CIT0013] Foster, K., Maybery, D., Reupert, A., Gladstone, B., Grant, A., Ruud, T. et al., 2016, ‘Family-focused practice in mental health care: An integrative review’, *Child & Youth Services* 37(2), 129–155. 10.1080/0145935X.2016.1104048

[CIT0014] Gaolaolwe, W., Manyedi, E. & Serapelwane, M., 2023, ‘Family members’ experiences of courtesy stigma associated with mental illness’, *Health SA Gesondheid* 28(1), 1–14. 10.4102/hsag.v28i0.2184 PMC10476503 37670747

[CIT0015] Grove, S.K., Burns, N. & Gray, J., 2021, *The practice of nursing research: Appraisal, synthesis and generation of evidence*, Elsevier Health Sciences, St. Louis.

[CIT0016] Hong, Q.N., Gonzalez-Reyes, A. & Pluye, P., 2018a, ‘Improving the usefulness of a tool for appraising the quality of qualitative, quantitative and mixed methods studies, the Mixed Methods Appraisal Tool (MMAT)’, *Journal of Evaluation in Clinical Practice* 24(3), 459–467. 10.1111/jep.12884 29464873

[CIT0017] Hong, Q.N., Pluye, P., Fàbregues, S., Bartlett, G., Boardman, F., Cargo, M. et al., 2018b, ‘Mixed methods appraisal tool (MMAT), version 2018’, Registration of copyright, 1148552(10).

[CIT0018] Hsiao, C.Y., Lu, H.L. & Tsai, Y.F., 2020, ‘Caregiver burden and health-related quality of life among primary family caregivers of individuals with schizophrenia: A cross-sectional study’, *Quality of Life Research* 29(10), 2745–2757. 10.1007/s11136-020-02518-1 32394137

[CIT0019] Hwang, J.J., Donnelly, T.T., Raffin Bouchal, S. & Davidson, S., 2023, ‘Factors influencing access to nonpharmacological interventions for community-dwelling seniors with mild-to-moderate dementia: An integrative review’, *Journal of Psychiatric and Mental Health Nursing* 30(6), 1054–1081. 37203563 10.1111/jpm.12932

[CIT0020] Iseselo, M.K., Kajula, L. & Yahya-Malima, K.I., 2016, ‘The psychosocial problems of families caring for relatives with mental illnesses and their coping strategies: A qualitative urban based study in Dar es Salaam, Tanzania’, *BMC Psychiatry* 16(1), 1–12. 10.1186/s12888-016-0857-y 27177934 PMC4867081

[CIT0021] Kahn, P.V., Wishart, H.A., Randolph, J.S. & Santulli, R.B., 2016, ‘Caregiver stigma and burden in memory disorders: An evaluation of the effects of caregiver type and gender’, *Current Gerontology and Geriatrics Research* 2016(1), 1–5. 10.1155/2016/8316045 PMC4749763 26941795

[CIT0022] Kugley, S., Wade, A., Thomas, J., Mahood, Q., Jørgensen, A.M.K., Hammerstrøm, K. & Sathe, N., 2016, ‘Searching for studies: A guide to information retrieval for Campbell’, *Campbell Systematic Reviews* 13(1), 1–73. 10.4073/cmg.2016.1

[CIT0023] Kumar, R. & Das, A., 2017, ‘Needs of caregivers of persons with mental illness: Rehabilitation perspective’, *Indian Journal Psychiatric Social Work* 8(2), 18–22, viewed 14 December 2023, from http://pswjournal.org/index.php/ijpsw/article/view/26.

[CIT0024] Lamb, H.R. & Weinberger, L.E., 2020, ‘Deinstitutionalization and other factors in the criminalization of persons with serious mental illness and how it is being addressed’, *CNS Spectrums* 25(2), 173–180. 10.1017/S1092852919001524 31599221

[CIT0025] Lawless, J. & Foster, M.J., 2020, ‘Searching systematically and comprehensively’, in C.E. Toronto & R. Remington (eds.), *A step-by-step guide to conducting an integrative review*, pp. 21–44, Springer International Publishing, Cham.

[CIT0026] Liu, Z., Heffernan, C. & Tan, J., 2020, ‘Caregiver burden: A concept analysis’, *International Journal of Nursing Sciences* 7(4), 438–445. 10.1016/j.ijnss.2020.07.012 33195757 PMC7644552

[CIT0027] Marimbe, B.D., Kajawu, L., Muchirahondo, F., Cowan, F. & Lund, C., 2016, ‘Perceived burden of care and reported coping strategies and needs for family caregivers of people with mental disorders in Zimbabwe’, *African Journal of Disability* 5(1), 1–9. 10.4102/ajod.v5i1.209 PMC5433451 28730046

[CIT0028] Mark, D.E., 2013, *Experiences of caregivers for relatives with a chronic severe mental illness: Riding the roller coaster: A descriptive study*, pp. 1–83, viewed 17 December 2023, from https://scholarworks.smith.edu/theses/1001.

[CIT0029] Moudatsou, M., Koukouli, S., Palioka, E., Pattakou, G., Teleme, P., Fasoi, G. et al., 2021, ‘Caring for patients with psychosis: Mental health professionals’ views on informal caregivers’ needs’, *International Journal of Environmental Research and Public Health* 18(6), 1–14. 10.3390/ijerph18062964 PMC8001319 33799346

[CIT0030] Molepo, M.M. & Mfidi, F.H., 2020, ‘Lived experiences of young people who live with mental healthcare users in Limpopo, South Africa’, *Africa Journal of Nursing and Midwifery* 22(2), 1–11. 10.25159/2520-5293/4713

[CIT0031] Nair, S., Jagannathan, A., Kudumallige, S., Kumar, C.N. & Thirthalli, J., 2018, ‘Need for micro-finance self-help groups among women family caregivers of persons with mental disability in rural India’, *Mental Health and Social Inclusion* 22(1), 34–45. 10.1108/MHSI-10-2017-0039

[CIT0032] Ndlovu, J.T. & Mokwena, K.E., 2023, ‘Burden of care of family caregivers for people diagnosed with serious mental disorders in a rural health district in Kwa-Zulu-Natal, South Africa’, *Healthcare* 11(19), 2686. 10.3390/healthcare11192686 37830723 PMC10572910

[CIT0033] Ntsayagae, E.I., Myburgh, C. & Poggenpoel, M., 2019, ‘Experiences of family caregivers of persons living with mental illness: A meta-synthesis’, *Curationis* 42(1), 1–9. 10.4102/curationis.v42i1.1900 PMC6779982 31588764

[CIT0034] O’Neill, S., Horigan, G., Gray, A.M., Gibson, S., Meehan, K. & Coates, V., 2022, ‘Women’s experiences when caring for relatives with mental illness in Northern Ireland: A qualitative study’, *Health & Social Care in the Community* 30(5), e2033–e2040. 10.1111/hsc.13637 34904317

[CIT0035] Ong, H.S., Fernandez, P.A. & Lim, H.K., 2021, ‘Family engagement as part of managing patients with mental illness in primary care’, *Singapore Medical Journal* 62(5), 213. 10.11622/smedj.2021057 34409463 PMC8801858

[CIT0036] Opondo, P.R., Olashore, A.A., Molebatsi, K., Othieno, C.J. & Ayugi, J.O., 2020, ‘Mental health research in Botswana: A semi-systematic scoping review’, *Journal of International Medical Research* 48(10), 1–16. 10.1177/0300060520966458 PMC7607297 33115301

[CIT0037] Page, M.J., McKenzie, J.E., Bossuyt, P.M., Boutron, I., Hoffmann, T.C., Mulrow, C.D. et al., 2021, ‘The PRISMA 2020 statement: An updated guideline for reporting systematic reviews’, *International Journal of Surgery* 134, 103–112. 10.1016/j.jclinepi.2021.02.003 PMC8008539 33781348

[CIT0038] Papaioannou, D., Sutton, A. & Booth, A., 2016, *Systematic approaches to a successful literature review*, pp. 1–336, SAGE, Los Angeles.

[CIT0039] Phoeun, B., Chanthorn, L., Schulhofer, L., Khann, S., Soung, T., Conroy, K. et al., 2023, ‘“I feel hopeless”: Exploring the psychosocial impacts of caring for mentally ill relatives in Cambodia’, *International Journal of Social Psychiatry* 69(2), 438–446. 10.1177/00207640221109171 35796433

[CIT0040] Rasmussen, N.L. & Montgomery, P., 2018, ‘The prevalence of and factors associated with inclusion of non-English language studies in Campbell systematic reviews: A survey and meta-epidemiological study’, *Systematic Reviews* 7(1), 1–12. 10.1186/s13643-018-0786-6 30139391 PMC6107944

[CIT0041] Shamsaei, F., Cheraghi, F. & Esmaeilli, R., 2015, ‘The family challenge of caring for the chronically mentally ill: A phenomenological study’, *Iranian Journal of Psychiatry and Behavioural Sciences* 9(3), 1–7. 10.17795/ijpbs-1898 PMC4644616 26576169

[CIT0042] Sharif, L., Basri, S., Alsahafi, F., Altaylouni, M., Albugumi, S., Banakhar, M. et al., 2020, ‘An exploration of family caregiver experiences of burden and coping while caring for people with mental disorders in Saudi Arabia – A qualitative study’, *International Journal of Environmental Research and Public Health* 17(17), 1–15. 10.3390/ijerph17176405 PMC7504338 32887502

[CIT0043] Sharma, N., Chakrabarti, S. & Grover, S., 2016, ‘Gender differences in caregiving among family-caregivers of people with mental illnesses’, *World Journal of Psychiatry* 6(1), 7. 10.5498/wjp.v6.i1.7 27014594 PMC4804270

[CIT0044] Siddiqui, S. & Khalid, J., 2019, ‘Determining the caregivers’ burden in caregivers of patients with mental illness’, *Pakistan Journal of Medical Sciences* 35(5), 1329–1333. 10.12669/pjms.35.5.720 31489001 PMC6717449

[CIT0045] Silaule, O., Gloria, N. & Adams, F., 2023, ‘Extent of caregiver burden among informal caregivers of persons with severe mental disorders in rural South Africa’, *Rural and Remote Health* 23(2), 1–12. 10.22605/RRH7509 37264594

[CIT0046] Silva, G.A.D., Cardoso, A.J.C., Bessoni, E., Peixoto, A.D.C., Rudá, C., Silva, D.V.D. et al., 2022, ‘Deinstitutionalization and autonomy: Outcomes from a Brazilian mental health policy’, *Ciência & Saúde Coletiva* 27, 101–110. 10.1590/1413-81232022271.19872021 35043890

[CIT0047] Smith, V., Devane, D., Begley, C.M. & Clarke, M., 2011, ‘Methodology in conducting a systematic review of systematic reviews of healthcare interventions’, *BMC Medical Research Methodology* 11(1), 15. 10.1186/1471-2288-11-15 21291558 PMC3039637

[CIT0048] Stanley, S., Balakrishnan, S. & Ilangovan, S., 2017, ‘Psychological distress, perceived burden and quality of life in caregivers of persons with schizophrenia’, *Journal of Mental Health* 26(2), 134–141. 10.1080/09638237.2016.1276537 28385096

[CIT0049] The Specialist Forum, 2020, ‘Caring for the caregivers’, *Psychiatry* 20(4), 12–15. 10.10520/ejc-nm_specf-v20-n4-a531918708

[CIT0050] Toronto, C.E. & Remington, R. (eds.), 2020, *A step-by-step guide to conducting an integrative review*, pp. 1–9, Springer International Publishing, Cham.

[CIT0051] Verity, F., Turiho, A., Mutamba, B.B. & Cappo, D., 2021, ‘Family care for persons with severe mental illness: Experiences and perspectives of caregivers in Uganda’, *International Journal of Mental Health Systems* 15(1), 1–9. 10.1186/s13033-021-00470-2 34016125 PMC8139105

[CIT0052] Von Kardorff, E., Soltaninejad, A., Kamali, M. & Eslami Shahrbabaki, M., 2016, ‘Family caregiver burden in mental illnesses: The case of affective disorders and schizophrenia – A qualitative exploratory study’, *Nordic Journal of Psychiatry* 70(4), 248–254. 10.3109/08039488.2015.1084372 26524243

[CIT0053] Vukeya, T., Temane, A. & Poggenpoel, M., 2022, ‘Experiences of family members caring for a sibling with mental illness in Giyani, Limpopo’, *Curationis* 45(1), 1–10. 10.4102/curationis.v45i1.2347 PMC9724046 36453820

[CIT0054] Werner, P., Mittelman, M.S., Goldstein, D. & Heinik, J., 2012, ‘Family stigma and caregiver burden in Alzheimer’s disease’, *The Gerontologist* 52(1), 89–97. 10.1093/geront/gnr117 22048807

[CIT0055] Whittemore, R. & Knafl, K., 2005, ‘The integrative review: Updated methodology’, *Journal of Advanced Nursing* 52(5), 546–553. 10.1111/j.1365-2648.2005.03621.x 16268861

[CIT0056] Zacharis, T., Lyrakos, G. & Zisi, V., 2020, ‘Physical activity and mental health in caregivers of mental ill patients in Greece’, *Journal of Human Sport and Exercise* 15(3proc), S848–S855. 10.14198/jhse.2020.15.Proc3.36

